# Draft Genome Sequence of Streptococcus suis S10, a Virulent Strain Used in Experimental Pig Infections

**DOI:** 10.1128/MRA.00227-19

**Published:** 2019-06-06

**Authors:** Rogier A. Gaiser, Aldert L. Zomer, Jerry M. Wells, Peter van Baarlen

**Affiliations:** a Host-Microbe Interactomics, Wageningen University & Research, Wageningen, The Netherlands; b Veterinary Medicine, Department of Infectious Diseases and Immunology, Utrecht University, Utrecht, The Netherlands; University of Maryland School of Medicine

## Abstract

Here, we report the draft whole-genome sequence of Streptococcus suis strain S10, isolated from the tonsils of a healthy pig. S. suis S10 belongs to the highly virulent serotype 2, which includes isolates that cause infectious diseases, including meningitis, in pigs and human. The genome contains a complete prophage that encodes a candidate virulence gene.

## Abstract (precis)

Here, we report the draft whole-genome sequence of Streptococcus suis strain S10, isolated from the tonsils of a healthy pig. S. suis S10 belongs to the highly virulent serotype 2, which includes isolates that cause infectious diseases, including meningitis, in pigs and human.

## ANNOUNCEMENT

Streptococcus suis bacteria are commonly part of the porcine tonsillar microbiota (1), comprising carriage strains and strains that cause infectious disease in pigs and humans (2). Here, we report the genome sequence of S. suis strain S10, sampled in 1992 from porcine tonsils, which has been used for experimental infections of pigs (3).

S. suis S10 was cultured without agitation at 37°C with 5% CO_2_ in Todd-Hewitt Broth (THB) (Oxoid, UK). Genomic DNA was extracted from 2 ml of exponentially growing bacteria that were lysed in Nuclei Lysis solution (Promega) with proteinase K and protein precipitation solution (Promega) following the manufacturer’s protocol. DNA was precipitated in isopropanol and purified using phenol-chloroform-isoamyl alcohol following the protocols described by Barker (4). DNA purity and quality were assessed by gel electrophoresis and spectrometric analysis (ND-1000; NanoDrop Technologies).

DNA library preparation using the Illumina Nextera XT kit following the manufacturer's protocol and genomic DNA sequencing using an Illumina HiSeq 2500 instrument on a paired-end library (125 cycles) were carried out at BaseClear B.V. (Leiden, The Netherlands). FASTQ sequence files were generated using the Illumina CASAVA pipeline 1.8.3. A BaseClear in-house pipeline carried out quality assessment of paired-end reads by Illumina Chastity filtering, removal of reads containing adapters, PhiX control signal, and FASTQC Quality Control Tool 0.10.0. CLC Genomics Workbench 8 trimmed low-quality bases and assembled reads into contigs. KmerGenie (5) determined the optimal k-mer size. SSPACE Premium Scaffolder 2.3 (6) linked contigs and placed them into scaffolds. GapFiller 1.10 (7) closed gapped regions within scaffolds. For all *in silico* analyses, default parameters were used.

Sequencing generated 4,268,836 paired-end reads (average length, 126 bases) from which 2,011,091 bases were aligned (266.38-fold coverage). Filtered reads were assembled in 32 gap-closed scaffolds. Quast (8) showed that the GC content was 41.25%, the *N*_50_ and *L*_50_ values were 170,355 and 5, and the *N*_75_ and *L*_75_ values were 86,491 and 9. The number of uncalled bases per assembled 100 kbp was 0.10.

The S10 genome was annotated via Prokka (9). ANItools Web (10) showed that S. suis strain P1/7 (11) was most identical to S10 (99.97%; not shown). CONTIGuator (12) aligned S10 genomic contigs to the P1/7 genome; 23 S10 contigs with a combined base content of 1,997,858 base pairs were mapped to the total of 2,007,491 base pairs of P1/7.

**FIG 1:**
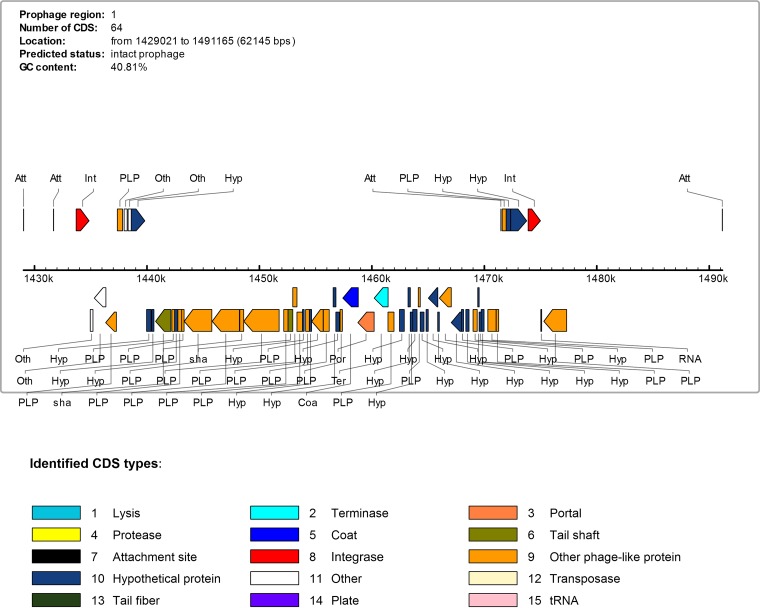
Phage Search Tool Enhanced Release (PHASTER) analysis output displaying the 64 coding sequences (CDS) of phage origin and their functional annotations; the annotated region shown is part of the largest S10 region that did not align to S. suis strain P1/7. Att, attachment site; Hyp, hypothetical protein; Int, integrase; Oth, other protein; PLP, phage-like protein.

In contig_00009, Phage Search Tool Enhanced Release (PHASTER) (13) predicted a complete prophage containing 64 coding sequences (CDS) spanning a region of 62,145 base pairs (Fig. 1). The predicted S10 phage contains a candidate virulence gene with high identity (E value, 7E−164) with the S. mitis (14) and S. pneumoniae (15) phage-encoded gene *pblB*, which encodes a platelet adhesin that plays a role during multiple steps in endocardial infection, including direct binding of bacteria to platelets.

The genome sequence of S. suis S10 will enable the research community to make targeted gene deletion mutants.

### Data availability.

The genome sequence of strain S. suis S10 has been deposited at the European Molecular Biology Laboratory, European Bioinformatics Institute (EMBL-EBI); the raw reads have been deposited at the European Nucleotide Archive (ENA) under the study identifier PRJEB30600.
